# Influence of interrupted versus continuous suture technique on intestinal anastomotic leakage rate in patients with Crohn’s disease — a propensity score matched analysis

**DOI:** 10.1007/s00384-022-04252-1

**Published:** 2022-10-10

**Authors:** Anke Mittelstädt, Tobias von Loeffelholz, Klaus Weber, Axel Denz, Christian Krautz, Robert Grützmann, Georg F. Weber, Maximilian Brunner

**Affiliations:** Department of General and Visceral Surgery, Friedrich-Alexander-University, Krankenhausstraße 12, Erlangen, Germany

**Keywords:** Intestinal anastomosis, Crohn’s disease, Continuous suture technique, Interrupted suture technique, Anastomotic leakage

## Abstract

**Purpose:**

Intestinal anastomosis is a crucial step in most intestinal resections, as anastomotic leakage is often associated with severe consequences for affected patients. There are especially two different techniques for hand-sewn intestinal anastomosis: the interrupted suture technique (IST) and the continuous suture technique (CST). This study investigated whether one of these two suture techniques is associated with a lower rate of anastomotic leakage.

**Methods:**

A retrospective review of 332 patients with Crohn’s disease who received at least one hand-sewn colonic anastomosis at our institution from 2010 to 2020 was performed. Using propensity score matching 183 patients with IST were compared to 96 patients with CST in regard to the impact of the anastomotic technique on patient outcomes.

**Results:**

Overall anastomotic leakage rate was 5%. Leakage rate did not differ between the suture technique groups (IST: 6% vs. CST: 3%, *p* = 0.393). Multivariate analysis revealed the ASA score as only independent risk factor for anastomotic leakage (*OR* 5.3 (95% *CI* = 1.2–23.2), *p* = 0.026). Suture technique also showed no significant influence on morbidity and the re-surgery rate in multivariate analysis.

**Conclusion:**

Our data suggest that the chosen suture technique (interrupted vs. continuous) has no influence on postoperative outcome, especially on anastomotic leakage rate. This finding should be confirmed by a randomized controlled trial.

## Introduction

Intestinal resections are one of the most common procedures in abdominal surgery. In the context of intestinal resections, intestinal anastomosis are of particular importance, as anastomotic leakage has far-reaching consequences for the affected patients including high re-surgery rates, higher risk of accompanying morbidities and longer hospital stays as well as impaired quality of life [1–6]. This aspect applies even more to patients with Crohn’s disease, as they have an increased risk of anastomotic leaks compared to patients without Crohn’s disease [7, 8].

There are different known risk factors associated with anastomotic leakage including aspects of surgical technique [7–11]. There are a number of technical variations for intestinal anastomosis regarding different aspects like kind of anatomical reconstruction (end-to-end vs. end-to-side vs. side-to-side), kind of suture techniques (stapled vs. hand-sewn, interrupted vs. continuous suture technique, single vs. double-layered technique) as well as the used suture material [12–14]. Therefore, the variability of the surgical technique for intestinal anastomosis may be one of the greatest among surgeons. And the “surgical school” may play a unique role for the surgical technique of the intestinal anastomosis.

In particular, there is a lack of sufficient evidence for the question of interrupted suture technique (IST) versus continuous suture technique (CST) [15]. Experimental investigations suggest that the interrupted suture technique might be associated with a better perfusion of anastomosis, whereas the continuous suture might offer a better sealing of the anastomosis [16]. Moreover, advocates of the continuous technique allege that the continuous suture may be able to save costs and operating time in comparison to the interrupted suture technique. However, until now there are only two retrospective studies that compared IST and CST with conflicting results. While a more recent study with 347 patients was able to show a significant difference between the suture techniques (IST: 16% vs. CST: 2.5%, *p* = 0,001), another investigation with only 53 patients showed no difference in the insufficiency rate (IST: 3.7% vs. CST: 3.8%) [10, 17].

Therefore, we focused on the influence of hand-sewn interrupted suture technique (IST) versus continuous suture technique (CST) on the postoperative outcome, especially on leakage rate and chose the high-risk population of patients with Crohn’s disease.

## Materials and methods

We retrospectively analyzed 332 consecutive patients with Crohn’s disease who received an intestinal resection and anastomosis from 2010 to 2020 at the University Hospital Erlangen. Patients had to meet the following inclusion criteria: (1) age greater than or equal to 18 years; (2) histologically proven Crohn’s disease; (3) at least one colonic anastomosis; (4) intestinal anastomosis in hand-sewn technique. Anastomoses with a protective stoma were excluded. Both open and laparoscopic approaches and both elective and emergency surgeries were allowed.

Data on patient demographics, comorbidities, preoperative parameters, and surgical technique as well as on the postoperative course including anastomotic leakage and morbidity were obtained and analyzed. Primary outcome was the occurrence of anastomotic leakage (see definition below). As secondary outcome influence of suture technique on morbidity (defined by Clavien Dindo [18]), wound healing CDC definition [19]) and re-surgery rate were investigated.

This study was approved by the Ethics Committee of FAU Erlangen (22–222-Br).

### Surgical techniques

All patients received preoperative intravenous antibiotic prophylaxis with a cephalosporin and metronidazole.

All intestinal anastomoses were performed based on three fundamental key points: (1) sufficient mobilization of the intestinal ends to obtain a tension-free anastomosis; (2) preservation of adequate blood perfusion of both intestinal ends; (3) low-bleeding preparation through subtle hemostasis.

Decision about anastomotic technique was made by the surgeon depending on his preference.

1. Interrupted suture technique (IST):
  This technique was always performed as single-layered anastomosis with extramucosal inverting stiches using 3/0 polyglactin (Vicryl, Ethicon).
2. Continuous suture technique (CST):
  For this technique single and double-layered technique was used. Suture material included 4/0 or 5/0 polydioxanone (PDS, Ethicon) and 4/0 polyglyconate (Maxon, Covidien).

### Definition of anastomotic leakage

Postoperative anastomotic leakage of the intestinal anastomosis was defined as the presence of at least one of the following criteria: (1) Evidence of anastomotic leakage by endoscopy; (2) radiological evidence of leakage by contrast-enhanced computer tomography; (3) evidence of leakage during re-surgery.

### Statistical analysis

For propensity score matching, the nearest neighbor method to 2:1 ratio was used. Propensity score deviation width was set to a threshold of < 0.2. Variables used for matching were age, gender, and surgically relevant factors: surgical priority and number of anastomosis. Data analysis was performed with SPSS software (SPSS, version 28.0). Comparisons of metric and ordinal data were calculated with the Student’s t-test or Mann–Whitney *U* test. The chi-square test was used for categorical data. Statistical significance was set at *p* < 0.05. All recorded parameters were tested as potential risk factors for postoperative outcome parameters (morbidity, anastomotic leakage, wound infection, re-surgery) using univariate analysis. Associations with the outcome parameters with a *p*-value ≤ 0.1 in univariate analysis were included in multivariate analysis.

## Results

### Demographics

Propensity score matching of the 332 patients (median age: 36 years, 49% female) meeting inclusion criteria revealed 279 matched patients. Of these 279 patients, interrupted suture technique was applied in 183 patients (IST group) and continuous suture technique in 96 patients (CST group). Nine patients of the CST group had only one matched partner in the IST group.

Patients of the IST group had significantly more previous surgeries (1 vs. 0, *p* = 0.008) and a significantly lower hemoglobin (12.8 vs. 13.4 g/dl, *p* = 0.042). All other demographic parameters including age, gender, ASA, comorbidities, and preoperative blood results other than hemoglobin did not significantly differ between the groups (Table 1).

**Table 1:** Patients’ characteristics

	All patients	Interrupted suture	Interrupted suture	P
**Number**	279	183	96	
Age* (years), median (IQR)	34 (22)	35 (21)	32 (24)	0.412
Gender*, *n* (%)				0.9
** Female**	141 (51)	93 (51)	48 (50)	
** Male**	138 (49)	90 (49	48 (50)	
ASA** (*n*=268				0.328
** I**	18 (7)	14 (8)	4 (4)	
** II**	222 (83)	140 (81)	82 (87)	
** III**	28 (10)			
**BMI (kg/m**^**2**^**), median (IQR)**	22.7 (6.4)	22.5 (6.3)	22.8 (7.8)	0.531
**Arterial hypertension,* n* (%)**	43 (15)	30 (16)	13 (14)	0.602
**Coronary heart disease, *n* (%)**	7 (3)	5 (3)	2 (2)	1.000
**Diabetes, *n* (%)**	7 (3)	5 (3)	2 (2)	1.000
**Smoking history, *n* (%)**	128 (46)	86 (47)	42 (44)	0.897
**Number of previous surgeries, median (IQR)**	1 (2)	1 (2)	0 (1)	**0.008**
**Duration Crohn`s disease (years), median (IQR)**	7 (13)	7 (16)	7 (13)	0.736
**Montreal classification for Crohn`s disease**				
** Age at diagnosis, *n* (%)**				0.542
** A1: Below 16 years**	43 (15)	25 (14)	18 (19)	
** A2: Between 17 and 40 years**	198 (71)	133 (73)	133 (73)	
** A3: Above 40 years**	38 (14)	25 (14)	13 (13)	
** Location, *n* (%)**				**0.039**
** L1: Ileal**	0 (0)	0 (0)	0 (0)	
** L2: Colonic**	17 (6)	16 (9)	1 (1)	
** L3: Ileocolonic**	167 (60)	99 (54)	68 (71)	
** L4: Isolated upper disease**	0 (0)	0 (0)	0 (0)	
** L1 and L2**	6 (2)	4 (2)	2 (2)	
** L1 and L3**	19 (7)	12 (7)	7 (7)	
** L2 and L3**	54 (19)	40 (22)	14 (15)	
** L1 and L2 and L3**	16 (6)	12 (7)	4 (4)	
** Behavior, *n* (%)**				
** B1: Non-stricturing, non-penetrating**	8 (3)	6 (3)	2 (2)	0.055
** B2: Stricturing**	215 (77)	133 (73)	82 (85)	
** B3: Penetrating**	56 (20)	44 (24)	12 (13)	
** p: perianal disease**	38 (14)	30 (17)	8 (8)	0.067
Oral anti-inflammatory and/or immunosuppressive medication***, *n* (%)	153 (56)	107 (59)	46 (50)	0.157
**Preoperative WBC (10**^**9**^**/l), median (IQR)**	8.8 (4.8)	9.1 (5.5)	8.7 (4.1)	0.25
Preoperative albumin (g/l)*, median (IQR)	39 (7)	39 (6)	39 (8)	0.661
**Preoperative CRP (mg/l), median (IQR)**	16 (51)	22 (56)	10 (35)	0.093
**Preoperative hemoglobin (g/dl), median (IQR)**	13.1 (2.5)	12.8 (2.6)	13.4 (2.1)	**0.042**
**Preoperative creatinine (mg/dl), median (IQR)**	0.8 (0.2)	0.8 (0.2)	0.8 (0.2)	0.841

*ASA* American Society of Anesthesiologists Score; *BMI* body mass index; WBC white blood cells; CRP C-reactive protein

^*^Matched parameter; **Data incomplete; ***within the last 12 weeks before surgery

### Characteristics of Crohn’s disease

At the time of surgery, Crohn’s disease had existed for an average of 7 years. Most patients were diagnosed with Crohn’s disease between the ages of 17 and 40 years (71%). The predominant pattern of Crohn’s disease was an ileocolic location (92%) and a stricturing behavior (77%). Fifty-six percent of the patients were treated with an oral anti-inflammatory and/or immunosuppressive therapy up to 12 weeks before surgery. Comparing Crohn’s characteristics between the suture technique groups, the only difference was that patients of the IST group had significantly more frequent colonic location of Crohn’s disease (40% vs. 22%, *p* = 0.039) (Table 1).

### Surgical parameters

Most patients underwent elective surgery (96%) and one intestinal anastomosis (88%; 226 ileo-colonic anastomosis, 19 colo-colonic anastomosis), whereas 12% received two (16 ileo-colonic + colo-colonic anastomosis, 12 ileo-colonic and small intestine anastomosis and 5 colo-colonic + small intestinine anastomosis) and 1 patient three intestinal anastomosis (colo-colonic + 2 × small intestine anastomosis). Sixty-five percent of all surgeries were performed open and 35% laparoscopically (Table 2). Significant differences between the IST and the CST groups regarding surgical parameters included a significantly more often open approach (79 vs. 38%, *p* < 0.001) in the IST compared to the CST group (Table 2).

**Table 2:** Surgical characteristics

	**All patients (*n* = 279)**	**Interrupted suture (*n* = 183)**	**Continuous suture (*n* = 96)**	***p***
Priority*, *n* (%) ** Elective** ** Emergency**	267 (96) 12 (4)	176 (96) 7 (4)	91 (95) 5 (5)	0.757
Number of anastomosis*, *n* (%) ** 1** ** 2** ** 3**	245 (88) 33 (12) 1 (0)	160 (87) 23 (13) 0 (0)	85 (89) 10 (10) 1 (1)	0.399
**Kind of anastomosis, *n* (%)** ** Ileo-colonic anastomosis** ** Colo-colonic anastomosis** ** Ileo-colonic and colo-colonic anastomosis** ** Colo-colonic and small intestinine anastomosis** ** Ileo-colonic and small intestine anastomosis** ** Colo-colonic and 2 × small intestine anastomosis**	226 (81) 19 (7) 16 (6) 5 (2) 12 (4) 1 (0)	142 (78) 18 (10) 11 (6) 4 (2) 8 (4) 0 (0)	84 (88) 1 (1) 5 (5) 1 (1) 4 (4) 1 (1)	0.050
**Surgical approach, *n* (%)** ** Open** ** Laparoscopic**	181 (65) 98 (35)	145 (79) 38 (21)	36 (38) 60 (63)	** < 0.001**
**Need of intraoperative blood transfusion, *n* (%)**	9 (3)	8 (4)	1 (1)	0.170
**Operative time (min), median (IQR)**	153 (60)	153 (56)	154 (64)	0.955

^*^Matched parameter

### Outcome parameters

Overall morbidity, respectively, mortality in our collective was 35% and, respectively, 0%. Most of the complications were minor complications (63%). Anastomotic leakage occurred in 5% and wound infection in 9%. Six percent of the patients required re-surgery. Reasons for re-surgery were anastomotic leakage (81%), small bowel perforation (13%), and hematoma of the abdominal wall (6%). Median length of postoperative stay was 7 days. Re-admission within 90 days was necessary in 9% of the patients (Table 3).

**Table 3:** Outcome parameters

	**All patients (*n* = 279)**	**Interrupted suture (*n* = 183)**	**Continuous suture (*n* = 96)**	***p***
***Primary outcome***				
** Anastomotic leakage, *n* (%)**	14 (5)	11 (6)	3 (3)	0.393
** Anastomotic leakage according to surgical approach, *n* (%)** ** Open (***n* = **181)** ** Laparoscopic (*n* = 98)**	13 (6) 1 (1)	11 (8) 0 (0)	2 (6) 1 (2)	0.745 1.000
***Secondary outcomes***				
** Morbidity, *n* (%)**	97 (35)	72 (39)	25 (26)	**0.034**
** Clavien-Dindo, *n* (%)** ** I** ** II** ** IIIa** ** IIIb** ** IV**	18 (7) 43 (15) 15 (5) 12 (4) 9 (3)	12 (7) 33 (18) 11 (6) 10 (6) 6 (3)	6 (6) 10 (10) 4 (4) 2 (2) 3 (3)	0.294
** Wound infection, *n* (%)**	25 (9)	19 (10)	6 (6)	0.280
** Re-surgery, *n* (%)**	16 (6)	14 (8)	2 (2)	0.062
** Reasons for re-surgery, *n* (%)** ** Anastomotic leakage** ** Small bowel perforation** ** Hematoma of the abdominal wall**	13 (81) 2 (13) 1 (6)	11 (79) 2 (14) 1 (7)	2 (100) 0 (0) 0 (0)	1.000
** Mortality**, *n* **(%)**	0 (0)	0 (0)	0 (0)	-
** Postoperative length of hospital stay (days), median (IQR)**	7 (4)	8 (5)	7 (3)	** < 0.001**
** Re-admission within 90 days, *n* (%)**	24 (9)	19 (10)	5 (5)	0.180

### Primary endpoint analysis

Anastomotic leakage rate did not differ significantly between the two groups (IST: 6% vs. CST: 3%, *p* = 0.393). Subgroup analysis with stratification of patients according to the surgical approach showed also no significant differences between the two suture technique groups (Table 3). In the univariate analysis, we identified eight risk factors for postoperative anastomotic leakage with a *p*-value ≤ 0.1 (higher ASA score, *p* < 0.001; higher number of previous surgeries, *p* = 0.003; age > 40 years at Crohn’s diagnosis, *p* = 0.043; increase of preoperative CRP, *p* = 0.046; emergency surgery, *p* = 0.081; open surgical approach, *p* = 0.054; higher number of anastomosis, *p* = 0.085; increase of operative time, *p* = 0.029). Among these variables, only an ASA score of III/IV (*OR* 5.32 (95% *CI* = 1.22–23.18), *p* = 0.026) was confirmed as independent risk factors for the development of anastomotic leakage in the multivariate analysis (Table 4).

**Table 4:** Uni- and multivariate analyses

	**No AL vs. AL**				**No morbidity vs. morbidity**				**No WI vs. WI**				**No re-surgery vs. re-surgery**			
	Uni-variate	Multivariate			Uni-variate	Multivariate			Uni-variate	Multivariate			Uni-variate	Multivariate		
		*OR*	95% *CI*	*p*		*OR*	95% *CI*	*p*		*OR*	95% *CI*	*p*		*OR*	95% *CI*	*p*
**Age** **(years)**	0.160	-	-	-	0.266	-	-	-	**0.048**	0.99	0.94–1.03	0.508	**0.010**	1.01	0.97–1.06	0.658
**Gender** **(male vs. female)**	0.120	-	-	-	0.590	-	-	-	0.790	-	-	-	0.329	-	-	-
**ASA** **(III/IV vs. I/II)**	** < 0.001**	**5.32**	**1.22–23.18**	**0.026**	**0.004**	2.07	0.79–5.44	0.140	**0.026**	1.51	0.41–5.53	0.533	** < 0.001**	**4.34**	**1.15–16.46**	**0.031**
**BMI (kg/m**^**2**^**)**	0.760	-	-	-	0.203	-	-	-	0.703	-	-	-	0.980	-	-	-
**Arterial hypertension** **(yes vs. no)**	0.534	-	-	-	0.465	-	-	-	0.078	1.18	0.30–4.71	0.813	0.288	-	-	-
**Coronary heart disease** **(yes vs. no)**	0.287	-	-	-	0.224	-	-	-	**0.007**	4.61	0.53–40.19	0.166	0.351	-	-	-
**Diabetes** **(yes vs. no)**	0.286	-	-	-	0.221	-	-	-	0.092	1.86	0.22–15.45	0.566	0.349	-	-	-
**Smoking history** **(yes vs. no)**	0.326	-	-	-	0.273	-	-	-	0.684	-	-	-	0.877	-	-	-
**Number of previous surgeries** **(*n*)**	**0.003**	1.29	0.93–1.78	0.131	0.056	1.03	0.85–1.25	0.760	** < 0.001**	**1.36**	**1.07–1.73**	**0.013**	** < 0.001**	1.23	0.95–1.58	0.118
**Duration Crohn’s disease** **(years)**	0.390	-	-	-	0.897	-	-	-	0.574	-	-	-	0.168	-	-	-
**Age at Crohn’s diagnosis** **(A1 vs. A2 vs. A3)**	**0.043**	-*	-*	0.433	0.221	-	-	-	**0.025**	-*	-*	**0.475**	**0.021**	-*	-*	0.381
**Location of Crohn’s disease** **(colonic involvement vs. no colonic involvement)**	0.183	-	-	-	0.329	-	-	-	0.108	-	-	-	0.366	-	-	-
**Behavior of Crohn’s disease** **(B1 vs. B2 vs. B3)**	0.577	-	-	-	**0.015**	-*	-*	0.106	**0.009**	-*	-*	0.092	0.719	-	-	-
Oral anti-inflammatory and/or immunosupp. medication** **(yes vs. no)**	0.326	-	-	-	0.896	-	-	-	0.547	-	-	-	0.389	-	-	-
**Preoperative WBC** **(10**^**9**^**/I)**	0.988	-	-	-	0.975	-	-	-	0.925	-	-	-	0.991	-	-	-
**Preoperative albumin** **(g/I)**	0.662	-	-	-	0.110	-	-	-	0.650	-	-	-	0.853	-	-	-
**Preoperative CRP** **(mg/l)**	**0.046**	1.04	1.00–1.01	0.413	0.108	-	-	-	0.386	-	-	-	0.245	-	-	-
**Preoperative hemoglobin** **(g/dl)**	0.213	-	-	-	** < 0.001**	**0.85**	**0.73–0.99**	**0.036**	0.443	-	-	-	0.078	0.94	0.66–1.33	0.725
**Preoperative creatinine** **(mg/dl)**	0.364	-	-	-	0.612	-	-	-	0.740	-	-	-	0.841	-	-	-
**Priority** **(emergency vs. elective)**	0.081	3.98	0,44–35.83	0.219	0.091	1.74	0.44–6.86	0.427	0.350	-	-	-	0.694	-	-	-
**Kind of anastomosis** **(at least one colo-colonic anastomosis vs. others)**	0.469	-	-	-	0.331	-	-	-	0.223	-	-	-	0.799	-	-	-
**Surgical approach** **(laparoscopic vs. open)**	0.054	0.43	0.04–4.15	0.463	** < 0.001**	0.52	0.26–1.05	0.069	**0.045**	0.57	0.16–1.94	0.364	**0.037**	0.28	0.03–2.74	0.275
**Suture technique** **(interrupted vs. continuous)**	0.303	-	-	-	**0.028**	1.44	0.74–2.81	0.288	0.256	-	-	-	0.076	2.07	0.38–11.31	0.401
**Number of anastomosis** **(*n*)**	0.085	2.60	0.66–10.28	0.174	0.502	-	-	-	0.598	-	-	-	0.459	-	-	-
**Operative time** **(min)**	**0.029**	1.01	1.00–1.02	0.126	** < 0.001**	**1.01**	**1.00–1.01**	**0.003**	**0.009**	1.00	1.00–1.01	0.321	** < 0.001**	**1.01**	**1.00–1.02**	**0.020**
**Need of intraoperative blood transfusion** **(yes vs. no)**	0.399	-	-	-	**0.019**	1.42	0.24–8.57	0.700	0.203	-	-	-	**0.050**	0.38	0.03–4.26	0.435

*ASA* American Society of Anesthesiologists Score; *BMI* body mass index; *WBC* white blood cells; *CRP* C-reactive protein

^*^No OR and no 95% *CI* specified, as three parameters were compared; ** within the last 12 weeks before surgery

### Secondary endpoint analysis

In univariate analysis, in-hospital-morbidity was significantly lower in the CST-group compared to the IST-group (CST: 26% vs. IST: 39%, *p* = 0.034). Stratified according to the Clavien Dindo classification, in-hospital morbidity did not differ between the groups (*p* = 0.294). Wound infection rate as well as the rate of re-surgery did not differ between the two groups (*p* = 0.280, respectively, *p* = 0.062) (Table 3).

In multivariate analysis, the suture technique could not be confirmed as independent risk factor for morbidity (*OR* 1.44 (95% *CI* = 0.74–2.81, *p* = 0.288). Independent risk factors for morbidity were a lower preoperative hemoglobin (*OR* 0.85 (95% *CI* = 0.73–0.99), *p* = 0.036) and a longer operative time (*OR* 1.01 (95% *CI* = 1.00–1.01), *p* = 0.003). A higher number of previous surgeries could be identified as an independent risk factors for wound infection (*OR* 1.36 (95% *CI* = 1.07–1.73), *p* = 0.013). There were two independent risk factors for the need for re-surgery: an ASA score of III/IV (*OR* 4.34 (95% *CI* = 1.15–16.46), *p* = 0.031) and a longer operative time (*OR* 1.01 (95% *CI* = 1.00–1.02), *p* = 0.020) (Table 4).

## Discussion

The surgical technique represents a decisive component for safe performance of intestinal anastomosis. Well-known surgical principles to prevent anastomotic leakage are gentle tissue handling, good hemostasis, adequate blood perfusion, asepsis, and a tension-free anastomosis. However, the evidence regarding interrupted versus continuous suture technique for intestinal anastomosis is insufficient.

The present study revealed no difference regarding the anastomotic leakage rate when comparing the interrupted and the continuous suture technique in the high-risk population of patients with Crohn’s disease. This result is in line with one of two existing previous studies with the same study endpoint. This study of Deen and Smart from 1995 was able to demonstrate an insufficiency rate of 3.8% and 3.7% for the interrupted and continuous suture technique, respectively, but in a clearly limited sample size of 57 patients [17]. In contrast, a more recent analysis of Eickhoff et al. from 2018 included 347 patients and could reveal a significant influence of the suture technique on anastomotic leakage showing a fivefold increased risk for anastomotic leakage using the interrupted suture technique (IST: 16% vs. CST: 2.5%, *p* = 0,001) [10]. However, one possible explanation for these ambiguous results might be the quite high anastomotic leakage rate of 16%, clustered especially in the group of interrupted suture technique, which the authors of the study have already mentioned as non-ideal anastomosis outcome.

In our cohort, the overall anastomotic leakage rate was 5% and is therefore in the range of those reported in the literature in which leakage rates vary between 1 and 10% for patients with different indications for surgery [6–8, 10, 11, 20–25]. Regarding patients with Crohn’s disease, an analysis of 463 patients with ileocolonic anastomosis using continuous suture technique by Volk et al. showed a leakage rate of 4.3% in this subgroup [8]. Repeated intestinal resection in patients with Crohn’s disease was associated with an increased rate of anastomotic leakage [24].

In our hospital, experiences with both techniques originate from different surgical schools. This fact could be a possible explanation for our result and may underline the importance of surgical school in intestinal anastomosis. Again, this suggests that surgeons should choose the technique with which they are used to and are more comfortable.

The only identified independent risk factor for anastomotic leakage in our cohort was the ASA score. This is in line with previous reports [6, 11, 20]. Moreover, in literature there are several other identified risk factors such as urgent operation setting, a body mass index > 25 kg/m^2^, diabetes mellitus, a hypotensive circulation upon admission, preoperative leukocytosis, intraoperative septic conditions, difficulties encountered during anastomosis, colocolic anastomosis, higher intraoperative blood loss, and postoperative blood transfusion [7, 8, 22, 23]. Some of these risk factors could not be confirmed, and some were not investigated in our study.

Secondary endpoint analysis showed that the suture technique has no relevant influence on morbidity, on wound infection rate as well as re-surgery rate in multivariate analysis. The significant association of morbidity with the suture technique in univariate analysis may be explained by the significant more often use of laparoscopic approach in continuous suture technique, which is known to be associated with less morbidity. The lack of impact of the suturing technique on the occurrence of wound infections was also demonstrated in the already mentioned study by Eickhoff et al. [10].

In our study, independent risk factors for morbidity were lower preoperative hemoglobin and longer operative time; for wound infection, a higher number of previous surgeries; and for re-surgery, an ASA score of III/IV and a longer operative time. All these are already reported risk factors in literature [8].

Our study is the first analysis using a propensity score matched cohort, which might be a relevant strength for homogenization of patient cohorts. Moreover, we selected the high risk population of patients with Crohn’s disease, which again leads to a homogenization of the patient population and gives our results additional relevance due to the increased insufficiency rates in this patient population. However, the present study has several limitations. First, the retrospective design of our study may have incurred some bias. Second, the patient cohort is heterogeneous regarding the surgical approach. Subsequently, the differences in univariate analysis for morbidity may be affected by the extended trauma in open surgery. However, the surgical approach was included to the multivariate analysis, so that a potential influence of this factor was taken into account.

## Conclusion

Our results show that in experienced hands, both the interrupted and the continuous suture technique can be performed with equal safety. Randomized controlled trials are needed to confirm these findings.

## Declarations

### Statement on the welfare of animals

This article does not contain any studies with animals performed by any of the authors.
